# Intraoperative Peripheral Intravenous Catheter Fracture During Modified Radical Neck Dissection

**DOI:** 10.7759/cureus.110016

**Published:** 2026-06-01

**Authors:** Çağdaş Baytar, Deniz Baklacı, Oguz Arslantürk

**Affiliations:** 1 Anesthesiology and Reanimation, Zonguldak Bülent Ecevit University, Zonguldak, TUR; 2 Otolaryngology, Bülent Ecevit University Faculty of Medicine, Zonguldak, TUR; 3 Cardiovascular Surgery, Zonguldak Bülent Ecevit University, Zonguldak, TUR

**Keywords:** catheter embolism, catheter fracture, intraoperative complication, patient safety, peripheral intravenous catheter, ultrasonography

## Abstract

Peripheral intravenous catheter (PIVC) fracture is a rare but potentially serious iatrogenic complication associated with intravascular fragment retention and embolic risk. We report a 67-year-old man undergoing modified radical neck dissection in whom the abrupt cessation of infusion revealed a fracture of an 18-gauge PIVC placed on the dorsum of the hand. Ultrasonography confirmed retained intravascular fragments. Surgical exploration under ongoing general anesthesia identified and successfully retrieved three fractured catheter segments within 10 minutes. The patient recovered uneventfully. This case emphasizes the importance of careful intraoperative catheter monitoring, awareness of mechanical compression risk -- including avoidance of placing a non-invasive blood pressure cuff on the same limb as the operative catheter -- and prompt imaging-guided management of suspected catheter fracture.

## Introduction

Peripheral intravenous catheters (PIVCs) are the most widely used vascular access devices in clinical practice, yet catheter fracture with intravascular embolization remains among their rarest and most serious complications [1,2]. In contrast to central venous catheter pinch-off syndrome, in which a subclavian catheter is compressed between the clavicle and the first rib [3], peripheral catheter fracture arises from mechanical stress, material fatigue, suboptimal insertion technique, or manufacturing defects [4,5]. A retained intravascular fragment may embolize distally or centrally, carrying the risks of vascular perforation, thrombophlebitis, septicemia, and cardiac arrhythmia [6].

These risks are amplified during prolonged head and neck surgery. Operative positioning typically requires both upper extremities to be tucked and circumferentially draped, immobilizing the cannulated limb in a fixed and frequently angulated posture for many hours and concealing the insertion site beneath the sterile field. Under these conditions, the catheter is exposed to sustained extrinsic compression and repetitive cyclic loading, while the direct visualization that would permit early recognition of infusion failure or catheter deformation is precluded. Despite this combination of mechanical vulnerability and diagnostic blindness, intraoperative PIVC fracture in this setting is rarely reported, and its clinical gravity may be underappreciated.

We present a case in which an 18-gauge PIVC fractured into three segments during a prolonged modified radical neck dissection, with the cannulated limb tucked beneath the drapes and a non-invasive blood-pressure (NIBP) cuff cycling repeatedly on the same arm. The retained fragments were localized by point-of-care ultrasonography and retrieved surgically within the same anesthetic episode. This case report follows the CAse REports (CARE) guidelines. Written informed consent was obtained from the patient for publication.

## Case presentation

A 67-year-old male patient (height: 172 cm, weight: 58 kg, and ASA physical status II) was scheduled for elective modified radical neck dissection by the otolaryngology-head and neck surgery team for a previously diagnosed malignancy. He had no significant comorbidities. Preoperative laboratory values and cardiopulmonary evaluation were within normal limits.

On arrival at the operating theater, the patient had a functioning 20-gauge PIVC in situ. Anesthetic induction was achieved with intravenous midazolam 1 mg, propofol 180 mg, fentanyl 100 mcg, and rocuronium 40 mg. Oral endotracheal intubation was performed uneventfully with an 8.0 mm cuffed endotracheal tube. Anesthesia was maintained with sevoflurane and a remifentanil infusion titrated to surgical stimulation. Standard monitoring included electrocardiography, NIBP, pulse oximetry, capnography, and neuromuscular function assessment; the NIBP cuff was placed on the left upper arm and cycled at regular intervals throughout the procedure.

Before surgical draping, an 18-gauge over-the-needle PIVC -- a fluorinated ethylene propylene (FEP) catheter, within its shelf life (expiry September 2028) -- was placed into the dorsum of the left hand. Insertion was achieved in a single pass, advanced smoothly without resistance, and at no point was the catheter withdrawn and re-advanced over the introducer needle. The catheter was secured with an adhesive dressing, and a 1000 mL bag of isotonic saline was connected and confirmed to run freely under gravity; no pressure bag or infusion pump was used. Both upper extremities were subsequently tucked and circumferentially draped beneath the surgical field as required for the operative position, leaving the cannulation site concealed and the NIBP cuff in place on the same (left) arm. The total duration of surgery was 5 hours and 50 minutes.

Intraoperative fluid management proceeded without incident through the first 1000 mL of isotonic saline, delivered by gravity via the newly placed 18-gauge catheter. During infusion of a second 1000 mL bag, flow ceased abruptly after approximately 200 mL had been administered. Because the cannulation site was inaccessible beneath the sterile drapes within the operative field, the catheter could not be directly inspected intraoperatively; ongoing fluid requirements were met through the pre-existing 20-gauge catheter. No attempt was made to forcibly flush or aspirate the dysfunctional catheter in order to avoid propelling any potential fragments toward the central circulation.

**Figure 1:**
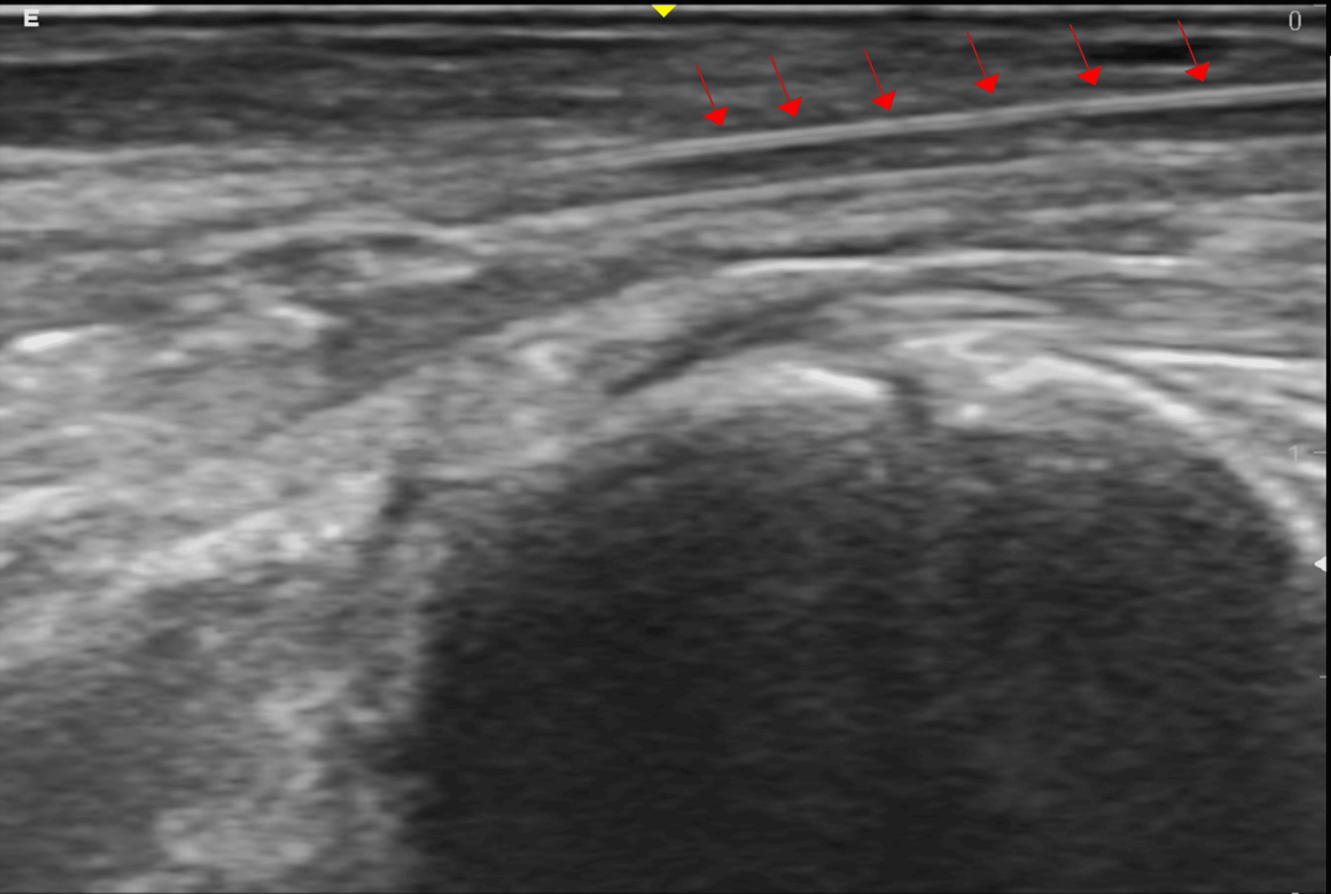
Ultrasonographic Visualization of a Retained Peripheral IV Catheter Fragment Point-of-care ultrasonographic image of the left hand dorsum demonstrating a hyperechoic linear intravascular structure (red arrows) consistent with a retained peripheral intravenous catheter fragment within the superficial dorsal hand vasculature. The echogenic appearance with posterior acoustic shadowing is characteristic of a retained polymer foreign body. The retained material appears as a single continuous structure because the fragments lay in close apposition within the same venous segment; their separation into three discrete pieces was resolved only at surgical exploration.

**Figure 2:**
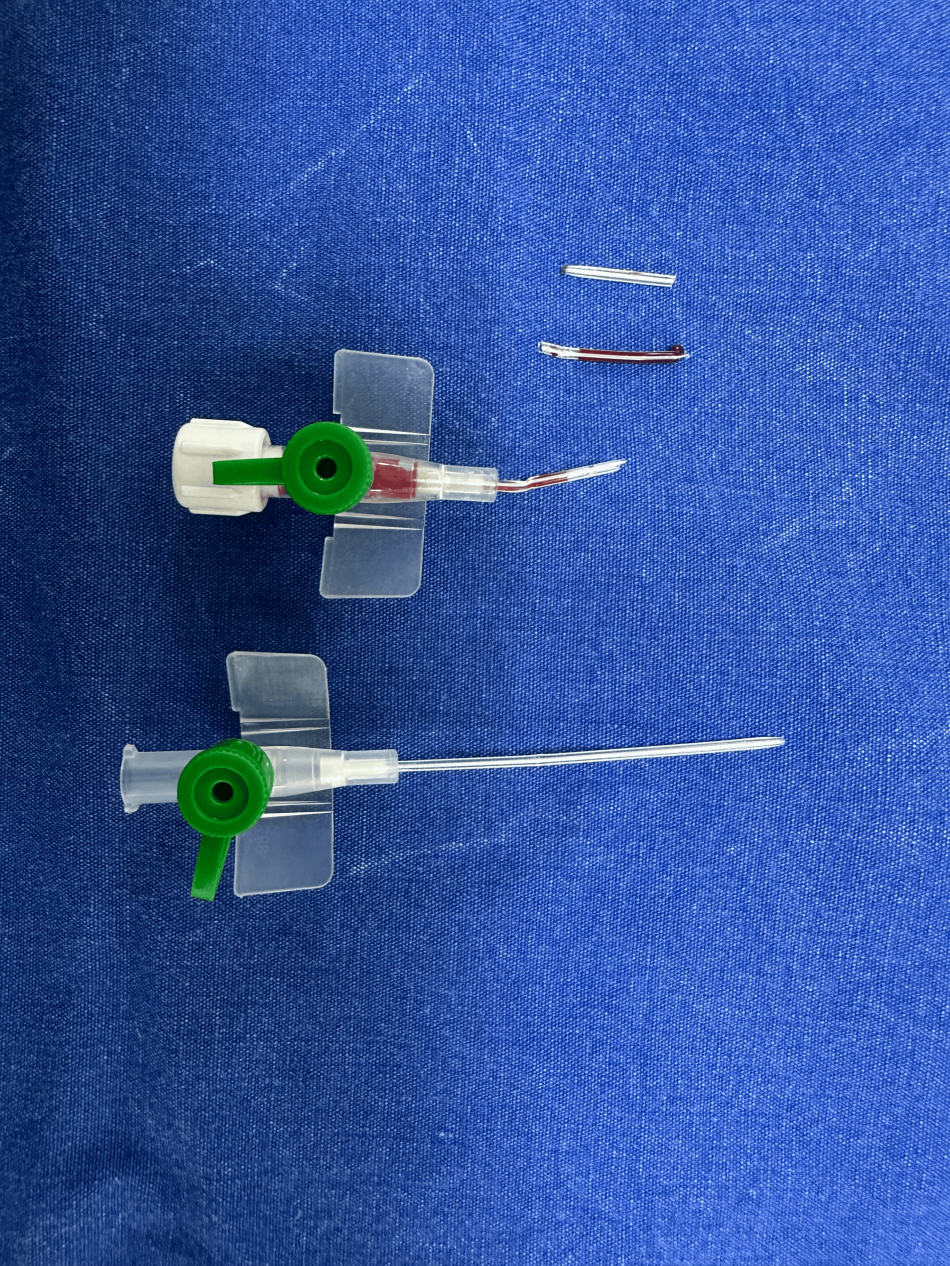
Fractured Peripheral Intravenous Catheter Following Removal From the Dorsal Hand Vein Ex vivo intraoperative photograph showing the fractured 18-gauge peripheral intravenous catheter after removal from the left hand dorsum. The upper device (fractured) is structurally incomplete, with the distal segment absent and the proximal hub-attached portion showing irregular fragmentation. The lower device is an intact 18-gauge catheter of the same model, included for comparison to demonstrate the normal catheter length and integrity.

**Figure 3:**
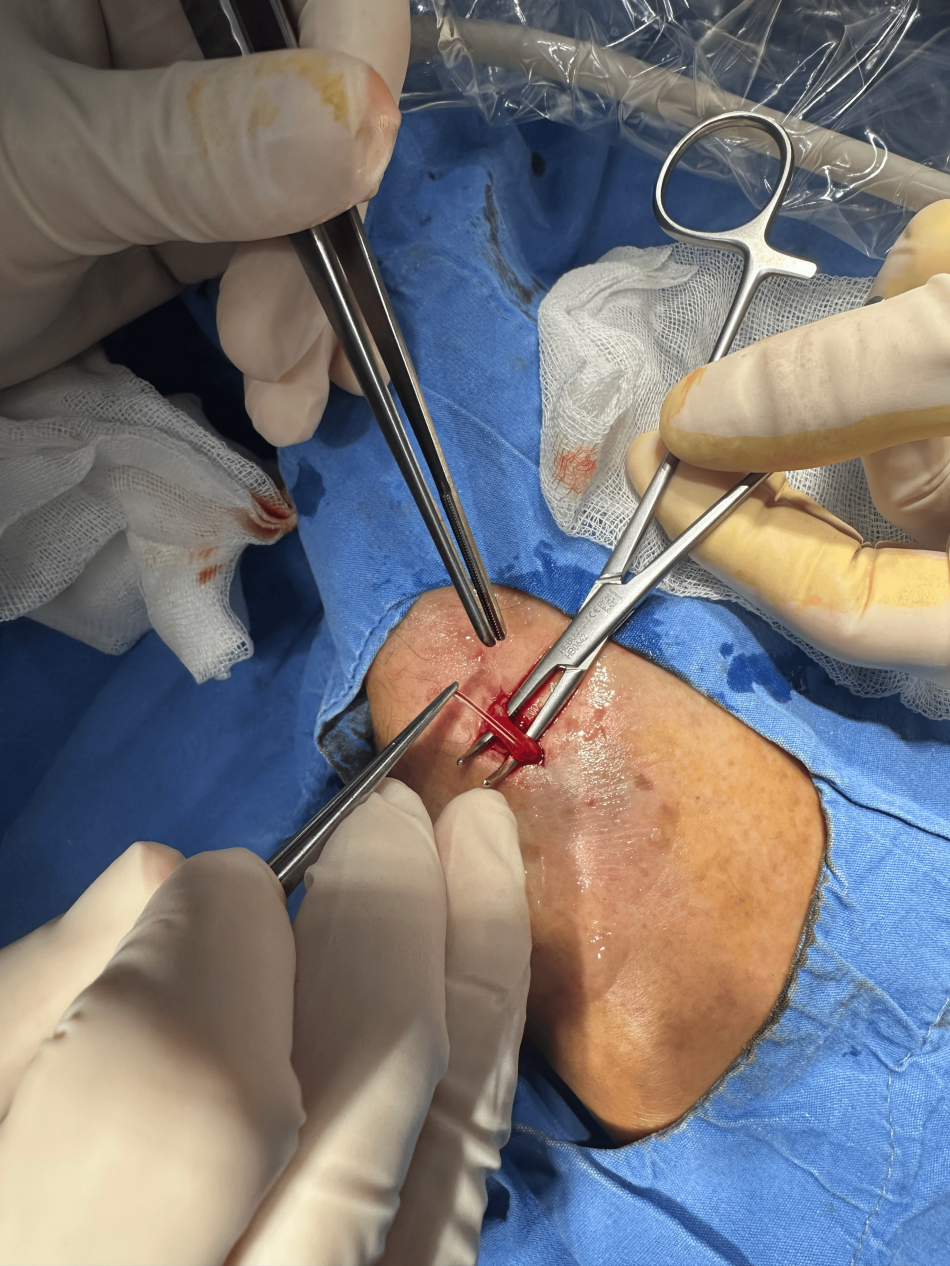
Surgical Retrieval of a Retained Peripheral IV Catheter Fragment From the Dorsal Hand Vein Intraoperative photograph depicting surgical exploration of the left hand dorsum under general anesthesia. A limited direct incision was made over the ultrasonographically localized fragment. The retained catheter segment is visible within the surgical field and is grasped with forceps before extraction. Fragment retrieval was completed within approximately 10 minutes while the patient remained intubated.

Upon completion of surgery and removal of the drapes, the non-functional 18-gauge PIVC was withdrawn as part of routine end-of-procedure care. Physical inspection of the retrieved cannula immediately revealed that it was structurally incomplete, with the distal portion absent -- indicating intravascular retention of at least one fragment. Mild dorsal hand edema with subcutaneous extravasation was noted clinically. Point-of-care ultrasonography performed at the bedside confirmed a retained hyperechoic linear structure within the dorsal hand vasculature (Figure 1). On ultrasonography, the retained material appeared as a single continuous hyperechoic structure, as the fragments lay in close apposition within the same short venous segment, and their separations were below the resolution achievable at the bedside. The cardiovascular surgery team was promptly consulted, and surgical retrieval was performed under the existing general anesthesia, as the patient had not yet been extubated. A direct incision was made over the ultrasonographically localized site, and on exploration, the catheter was found to have fractured into three discrete segments -- a proximal hub-attached segment, an intermediate shaft fragment, and a distal tip fragment (Figures 2, 3). All pieces were retrieved through a single incision within approximately 10 minutes.

The patient was subsequently extubated uneventfully. Postoperative hand function, perfusion, and neurological status were fully preserved, and the patient was discharged without sequelae.

## Discussion

This case illustrates a distinctive and largely preventable pattern of intraoperative PIVC fracture resulting in multi-fragment intravascular retention. Over-the-needle PIVCs are manufactured from flexible, fatigue-resistant polymers -- typically FEP or polyurethane -- yet these materials may still develop fatigue fractures when subjected to repetitive flexion, compression, or torsional loading [7]. Two features of this case are mechanistically relevant. First, the cannulated limb was tucked and circumferentially draped in a fixed, angulated position for nearly six hours, imposing sustained extrinsic compression on the catheter. Second, and we believe most importantly, the NIBP cuff cycled repeatedly on the same arm throughout the procedure; over an operation of this duration, this generates a large number of suprasystolic compression cycles transmitted along the limb, providing a plausible source of the cyclic mechanical loading required to propagate a fatigue fracture through an otherwise flexible catheter shaft. The abrupt cessation of infusion flow mid-bag is consistent with sudden structural failure rather than progressive occlusion.

Several alternative explanations were considered. Needle shearing at insertion was regarded as highly unlikely, as the cannula was placed in a single smooth pass without resistance and was never re-advanced over the needle. The device was within its shelf life (expiry September 2028), which argues against material degradation due to expiry. Nevertheless, the precise mechanism underlying the three-fragment pattern could not be definitively established, and intrinsic material variability or a manufacturing defect cannot be excluded. Formal device or material failure analysis was not performed, which we acknowledge as a limitation; for this reason, and given the medico-legal implications of attributing failure to a specific product without such analysis, the manufacturer and model are not identified here. Institutions encountering suspected device-related failures are encouraged to report them confidentially to the manufacturer and the relevant regulatory authority, as post-market surveillance data are essential for detecting batch-level patterns of structural failure [8].

Point-of-care ultrasonography proved invaluable in this case. As Kumar et al. have emphasized, ultrasound is the simplest and most accessible imaging modality for bedside localization of a fractured intravenous cannula [9]. Plain radiography and fluoroscopy may miss non-radiopaque polymer fragments, whereas ultrasound reliably demonstrates the characteristic hyperechoic linear appearance with posterior acoustic shadowing [10]. In this case, the three contiguous fragments were visualized as a single continuous hyperechoic structure; their discrete configuration became apparent only on surgical exploration -- a correspondence that clinicians should anticipate when comparing sonographic with operative findings.

The fortuitous circumstance that the patient remained intubated at the time of detection allowed expeditious surgical retrieval within the same operative episode, avoiding a second anesthetic induction. For fragments retained in distal extremity veins without evidence of proximal migration, direct surgical excision over the localized site is preferred to endovascular retrieval [11]. Forceful flushing of a potentially fractured catheter must be avoided, as it may propel retained fragments toward the central circulation [12]. From a preventive standpoint, the earliest recognizable warning sign in this case was the abrupt, mid-bag cessation of gravity infusion flow in a draped extremity; in the appropriate context, this should prompt suspicion of catheter structural failure rather than simple positional occlusion. Where feasible, the NIBP cuff and the operative intravenous catheter should not be placed on the same limb, and any cannulation site that will be inaccessible during surgery should be confirmed patent before final draping, with an alternative accessible access site available.

## Conclusions

PIVC fracture with intravascular fragment retention is a rare but serious intraoperative complication that may go unrecognized when the cannulation site is concealed beneath surgical drapes. In this case, prolonged positioning beneath drapes together with repetitive NIBP cuff compression on the same limb provided a plausible substrate for a multi-fragment fracture. Point-of-care ultrasonography offers rapid and reliable localization of retained fragments and should be the first-line imaging tool in this setting; when the complication is identified while the patient remains anesthetized, immediate coordination with surgical colleagues permits definitive retrieval within the same operative episode. Anesthesiologists should maintain a high index of suspicion when intraoperative infusion failure occurs in a draped extremity, avoid forceful catheter manipulation before a full assessment, and, where possible, avoid placing the NIBP cuff on the same limb as the operative intravenous catheter.
